# Separate and combined *Hanseniaspora uvarum* and *Metschnikowia pulcherrima* metabolic volatiles are attractive to *Drosophila suzukii* in the laboratory and field

**DOI:** 10.1038/s41598-020-79691-3

**Published:** 2021-01-13

**Authors:** R. Jones, M. T. Fountain, C. S. Günther, P. E. Eady, M. R. Goddard

**Affiliations:** 1 School of Life Sciences, University of Lincoln, Lincoln, LN6 7DL UK; 2 NIAB EMR, New Road, East Malling, Kent, ME19 6BJ UK; 3 The School of Biological Sciences, University of Auckland, Auckland, New Zealand; 4 Present Address: The New Zealand Institute of Plant and Food Research Ltd, Albert Road, Auckland, New Zealand

**Keywords:** Microbiology, Ecology, Ecology, Behavioural ecology

## Abstract

*Drosophila suzukii* flies cause economic losses to fruit crops globally. Previous work shows various *Drosophila* species are attracted to volatile metabolites produced by individual fruit associated yeast isolates, but fruits naturally harbour a rich diversity of yeast species. Here, we report the relative attractiveness of *D. suzukii* to yeasts presented individually or in combinations using laboratory preference tests and field trapping data. Laboratory trials revealed four of 12 single yeast isolates were attractive to *D. suzukii*, of which *Metschnikowia pulcherrima* and *Hanseniaspora uvarum* were also attractive in field trials. Four out of 10 yeast combinations involving *Candida zemplinina*, *Pichia pijperi*, *M. pulcherrima* and *H. uvarum* were attractive in the laboratory. Whilst a combination of *M. pulcherrima* + *H. uvarum* trapped the greatest number of *D. suzukii* in the field, the efficacy of the *M. pulcherrima* + *H. uvarum* combination to trap *D. suzukii* was not significantly greater than traps primed with volatiles from only *H. uvarum*. While volatiles from isolates of *M. pulcherrima* and *H. uvarum* show promise as baits for *D. suzukii*, further research is needed to ascertain how and why flies are attracted to certain baits to optimise control efficacy.

## Introduction

*Drosophila suzukii* (Matsumra), also known as spotted wing drosophila, is a damaging polyphagous pest that caused $511.3 million USD losses in three USA states in 2008 alone^1^. *D. suzukii* originated in Southeast Asia but has spread around the globe in the last 15 years and is now present in most Northern Hemisphere temperate regions. *D. suzukii* was first detected in Europe and mainland USA in 2008 and the UK in 2012^1–3^. Unlike other *Drosophila* species, *D. suzukii* can oviposit in a wide range of ripening fruits due to the females’ morphologically modified ovipositor which punctures the fruit epicarp to insert eggs^4^. Larvae feed inside the damaged fruits and exacerbate secondary infections by various microbes which further increase oviposition by other *Drosophila* species^5^, resulting in significant commercial losses. The main control measure for this pest is the application of insecticides coupled with crop hygiene and insect exclusion meshes. However, the use of insecticides is undesirable as these are not *D. suzukii* specific and have withholding (harvest interval) periods; in addition, the use of insecticides will select for resistance^6^. It is therefore valuable to evaluate complementary low or no-input methods that may be integrated with existing management options to control the burden of *D. suzukii* in fruit production.

Yeasts provide a source of protein for *Drosophila* which is important for fecundity and egg development^7,8^. *D. melanogaster* larvae fed on *Saccharomyces cerevisiae* yeast had faster development times and greater success in pupal development compared to flies on yeast-free diets^9^. *D. melanogaster* larval development time is more rapid on diets with greater yeast species diversity^10^. *D. suzukii* larvae survival is lower when reared on diets lacking yeast^11,12^ and a greater number of larvae survived when fed *Hanseniaspora uvarum* as opposed to *Metschnikowia pulcherrima* yeast^13^. Lewis and Hamby^12^ showed that while *D. suzukii* larvae exhibited a preference for *H. uvarum* over *S. cerevisiae* yeast, this preference did not translate to more rapid development time^12^*.* Given the apparent role of yeast in *Drosophila* fitness it is reasonable to suggest that natural selection will have operated on *Drosophila* traits that increase the probability of locating yeast laden-fruits^14,15^. In line with this prediction *Drosophila* females prefer to oviposit on yeast-colonised fruit^13^, and yeast metabolic volatiles act as cues for *Drosophila* to locate fruit with yeasts (e.g.^14,16^). The attraction of various *Drosophila* species to volatiles produced by different yeast species and strains within species varies^14,15,17,18^. *D. melanogaster* is attracted to a wide range of yeast from diverse backgrounds^19^ with a preference for *Saccharomyces* yeasts and species isolated from fruit^17^. Less is known for *D. suzukii*, but there are reports that isolates of *H. uvarum*, *Candida zemplinina*, *S. cerevisiae*, *Pichia terricola* and *Candida californica* yeasts are attractive to *D. suzukii*^18^.

Chandler et al*.*^20^ evaluated yeast communities from 11 different *Drosophila* species and showed the same yeast species tend to be associated with *Drosophila* irrespective of diet, *Drosophila* species or geographic location. *H. uvarum*, *Hanseniaspora valbenysis*, *M. pulcherrima* and *Torulaspora delbruckii* were associated with *D. simulans* and *D. melanogaster* from two vineyard sites in Australia^21^, and *H. uvarum* was the dominant yeast isolated from *D. suzukii* larvae and adults from US cherry orchards, followed by *Issatchenkia terricola* and *Pichia kluyveri*^22^. These species, among others, were also recovered from the larval frass of *D. suzukii,* again with *H. uvarum* being the most abundant^23^. *Hanseniaspora, Pichia* and *Candida* yeast genera were among those identified from the guts of wild caught winter-morph *D. suzukii* in the UK^24^. It thus appears that *H. uvarum* is often found associated with *D. suzukii;* however, *H. uvarum* is also very common on fruits including cherries, raspberries^22^, apples, plum, pears^25^ and grapes^26^ and so this association may simply reflect the fact that insects visiting fruits pick up common yeast as they do. Further, while specific *H. uvarum* isolates are attractive to specific *D. suzukii* lines in laboratory studies^18,27,28^, *H. uvarum* is also attractive to other *Drosophila* species, including *D. melanogaster*^15, 17,18,29^. Together this suggests that the *D. suzukii: H. uvarum* relationship is not specific.

Yeasts are single-celled fungi and while they are ubiquitous in terrestrial and aquatic habitats, they are commonly found associated with fruits (e.g.^30,31^). Yeasts have long been known to interact with some species of *Drosophila* via fruits^32^ and can be vectored by insects to new habitats^14,16^. Fruits are an ephemeral habitat which suggests natural selection will have operated on yeast traits that increase their likelihood of escaping fruits to colonise new habitats as this increases fitness; attracting vectors such as *Drosophila* is one way to achieve this^14,16^. There is some evidence for a mutualistic relationship between certain *Drosophila* and yeast isolates; for example, a more attractive *S. cerevisiae* isolate was vectored further by *D. simulans* than a less attractive one in laboratory and vineyard experiments, and *D. simulans* associated with the more attractive yeast also laid more eggs^14^. However, there is a lack of robust evidence to determine whether such interactions are driven by coevolution or due to exaptation of a coincidental combination of complementary traits^33^. Yeast metabolic volatile compounds can diffuse through both yeast cell walls and air and appear to mediate *Drosophila* attraction, but the nature of chemical attraction is complex as it appears concentration, background^34^ and substrate dependent^35^.

A range of yeast species are associated with fruits (e.g.^30,31^) and the particular mix of species in communities varies with geographic location^30,36,37^*,* ripening stage^26,38^, and fruit variety^36,39^. A recent experimental study showed that *Drosophila* attraction to individual yeasts can be unstable and evolve rapidly, suggesting instead that *Drosophila* may be generally adapted to sense and locate fruits infested by a community (i.e. a mix) of yeast species^15^. However, to date only single isolates of yeasts have been tested for *Drosophila* attraction (e.g.^14,17,18,34^). Here we investigate the attractiveness of both single yeast isolates and combinations of yeasts in laboratory and field assays to evaluate their potential as baits for use in integrated pest management strategies to control *D. suzukii*. Specifically, we test three main hypotheses: (1) some yeast isolates produce specific metabolic volatiles that are more attractive to *D. suzukii* than other *Drosophila* species*;* (2) volatiles from different genotypes (isolates) of *H. uvarum* vary in their attractiveness to *D. suzukii*; and (3) combinations of volatiles from different yeast isolates are more attractive to *D. suzukii* than volatiles from single yeasts. We evaluated the preference of *D. suzukii* under both laboratory and field conditions in order to ascertain the extent to which laboratory preferences predict the behaviour of *D. suzukii* in the field.

## Results

**Figure 1:**
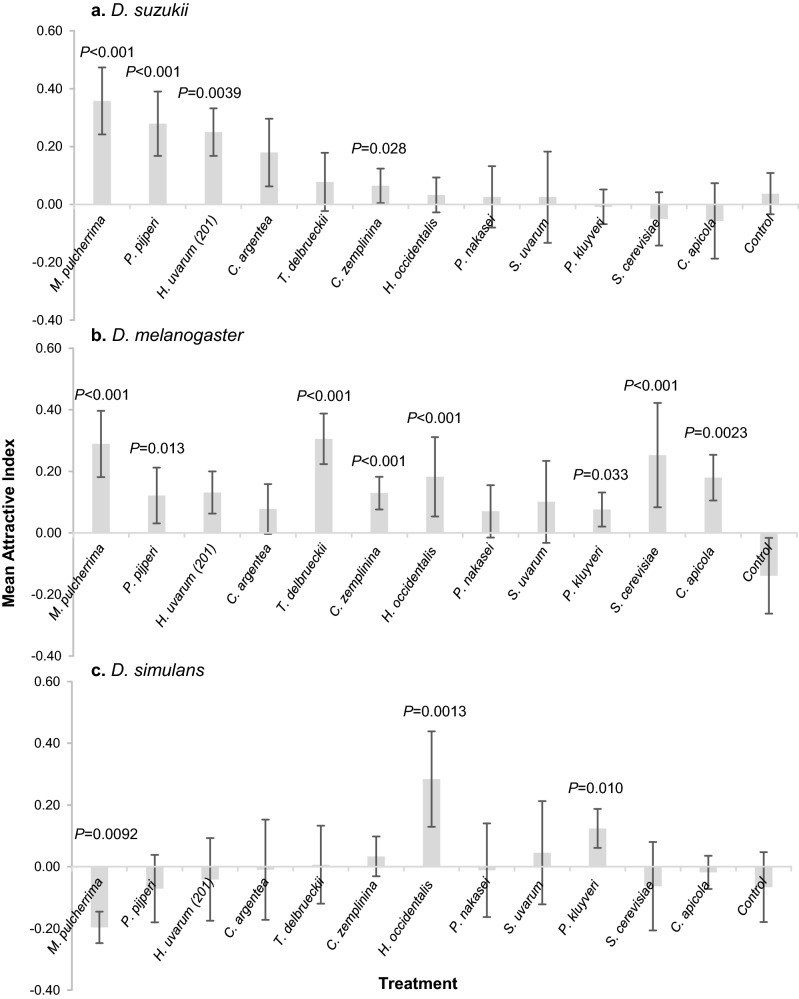
Mean (± SE) Attraction Index (AI) of *Drosophila* to metabolic volatiles (not identified) from single yeast isolates grown in sterile strawberry juice using laboratory T-mazes; control contains no yeast. (**a**) *D. suzukii* (N = 6 except *P. kluyveri* and *C. zemplinina* N = 18, N = 9 control)*.* (**b**) *D. melanogaster* (N = 6 except *P. kluyveri* N = 28 and *C. zemplinina* N = 24, control N = 9)*.* (**c**) *D. simulans* (N = 6 except *P. kluyveri*, *C. zemplinina* N = 18 and control N = 8). P-values above bars show significant attraction or repulsion to yeast volatiles over sterile strawberry juice in the opposing T-maze arm revealed by binomial analyses. The numbers of individuals that remained in the central compartment of the T-maze apparatus differed significantly between *Drosophila* species (binomial logistic regression, Δ deviance = − 741.03, df = − 2, *P* < 0.001; mean 69% *D. suzukii*, 42% *D. melanogaster* and 62% *D. simulans*).

We first tested 12 single yeast isolates separately for attractiveness to *D. suzukii, D. melanogaster* and *D. simulans* using laboratory T-maze choice experiments. Sterile fruit juice vs. sterile fruit juice controls showed no inherent bias in the T-maze apparatus for any fly species (*D. suzukii P* = 0.44; *D. melanogaster P* = 0.29, *D. simulans P* = 0.26; Fig. 1). *D. suzukii* displayed significant differential attraction to the yeast isolates (binomial logistic regression, Δ deviance = − 25.63, df = − 12, *P* = 0.012, Fig. 1a) and individual binomial analyses of fly numbers attracted to each yeast isolate showed four were significantly more attractive to *D. suzukii* compared to sterile strawberry juice and, in decreasing order of attraction, these were: *M. pulcherrima* (AI = 0.36, *P* = 0.0005), *P. pijperi* (AI = 0.28, *P* = 0.0006), *H. uvarum* 201 (AI = 0.25, *P* = 0.0039), and *C. zemplinina* (AI = 0.06 *P* = 0.028). *D. suzukii* showed neither preference nor repulsion to the remaining eight yeasts with binomial tests (Fig. 1a). *D. melanogaster* was also differentially attracted to the yeast isolates (Δ deviance = − 27.20, df = − 12 *P* = 0.0072; Fig. 1b). Like *D. suzukii, D. melanogaster* was attracted to *M. pulcherrima* (AI = 0.29, *P* = 0.00009), *C. zemplinina* (AI = 0.13, *P* = 0.00005), and *P. pijperi* (AI = 0.12, *P* = 0.013), but was additionally attracted to *T. delbrueckii* (AI = 0.31, *P* = 0.000005), *S. cerevisiae* (AI = 0.25, *P* = 0.0008), *H. occidentalis* (AI = 0.18, *P* = 0.0006), *C. apicola* (AI = 0.18, *P* = 0.0023), and *P. kluyveri* (AI = 0.09, *P* = 0.033). The remaining four yeast isolates were neither attractive nor repulsive to *D. melanogaster* (Fig. 1b). *D. simulans* was also differentially attracted to the yeast isolates (Δ deviance = − 21.52, df = − 12 *P* = 0.043, Fig. 1c), and individual binomial analyses suggest two of the yeasts were significantly attractive (*H. occidentalis* AI = 0.28, *P* = 0.0013 and *P. kluyveri* AI = 0.12, *P* = 0.010), but *M. pulcherrima* was significantly repulsive to *D. simulans* (AI = − 0.20, *P* = 0.0092). Taking all the data together, there was a significant effect of fly species (ANOVA, *F*_2,291_ = 3.77; *P* = 0.024) but not yeast species (ANOVA, *F*_12,291_ = 0.95; *P* = 0.49) on attraction, with no significant interaction between fly and yeast species (ANOVA, *F*_24,291_ = 1.45; *P* = 0.084). No one yeast isolate was significantly attractive to all three *Drosophila* species and *H. uvarum* 201 was the only yeast isolate to be significantly attractive to only *D. suzukii* (Fig. 1). However, we note that *H. uvarum* has been shown to be significantly attractive to *D. melanogaster* in a different study^15^.

### The attraction of *D. suzukii* to different isolates of *H. uvarum*

**Figure 2:**
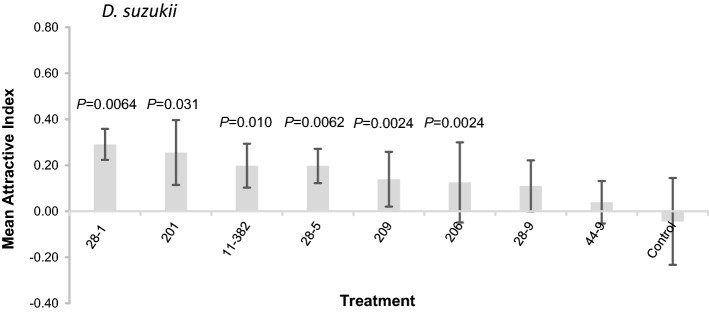
Mean (± SE) Attraction Index (AI) of *D. suzukii* to metabolic volatiles (not identified) from eight *Hanseniaspora uvarum* isolates grown in sterile strawberry juice using laboratory T-mazes; control contains no yeast (N = 8, except 28–1 and control, N = 7). P-values above bars show significance of attraction to yeast volatiles over sterile strawberry juice in the opposing T-maze arm revealed by binomial analyses.

There was no significant difference in attraction of *D. suzukii* to eight *H. uvarum* isolates (binomial logistic regression, Δ deviance = − 4.98, df = − 8, *P* = 0.76; Fig. 2). However, individual binomial tests indicate six isolates were more attractive than sterile fruit with *P* < 0.05, which includes the 201 isolate used above, and the 11-382 isolate shown to be attractive to *D. suzukii* previously^28^. The remaining two isolates were neither attractive nor repulsive compared to sterile strawberry juice.

### *D. suzukii* attraction to single yeasts in the field

**Figure 3:**
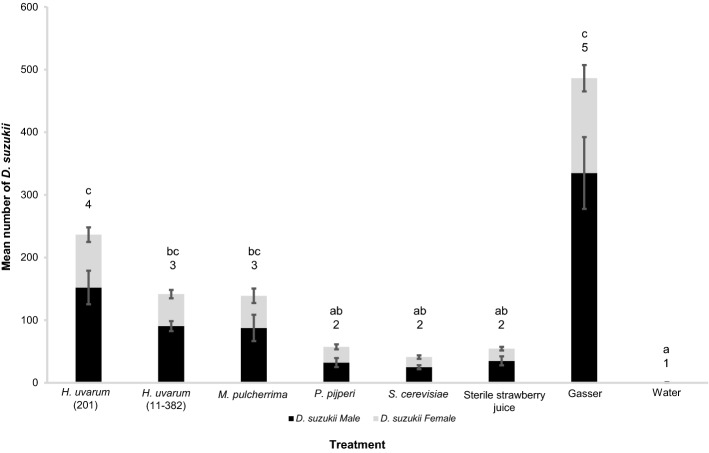
Mean numbers (± SE) of *D. suzukii* caught in field traps over a 72-h period baited with metabolic volatiles (not identified) from five different yeasts after individual growth in strawberry juice; sterile strawberry juice, water and commercial Gasser-lure are controls (N = 6). There was a significant effect of bait type on numbers of *D. suzukii* caught (linear model Δ deviance = 6434, df = 7, *P* < 0.001; Kruskal–Wallis, chi-squared = 41.59, df = 7, *P* = 0.62^–7^). Two post-hoc analysis methods were employed: letters above bars connect treatments that are not significantly different using Dunn’s comparisons with Benjamini–Hochberg adjusted *P* values, and numbers above bars connect treatments with overlapping 95% confidence intervals calculated from Estimated Marginal Means.

Yeast isolates more attractive to *D. suzukii* than sterile fruit in laboratory tests were taken forward to field assays along with commercially available “Gasser” (RIGA) traps and the *S. cerevisiae* isolate as this was not attractive to *D. suzukii* but was attractive to *D. melanogaster* in laboratory tests. The majority of the 8,400 *Drosophila* trapped were *D. suzukii* (83%) with the remainder mostly from the *D. obscura/subobscura* species group. There was a significant effect of bait type on numbers of *D. suzukii* trapped (linear model Δ deviance = 6434, df = 7, *P* < 0.001; Kruskal–Wallis, chi-squared = 41.59, df = 7, *P* = 0.62^–7^; Fig. 3). Gasser-lure trapped the greatest numbers of *D. suzukii* but Dunn’s comparisons post-hoc (with Benjamini–Hochberg multiple comparison correction) showed these were not significantly greater than numbers trapped by *H. uvarum* 201, *H. uvarum* (11-382) and *M. pulcherrima* yeasts (Fig. 3). However, marginal means analyses evaluating 95% confidence limits from the linear model indicate that Gasser-lure trapped significantly greater numbers of *D. suzukii*. When the effect of yeasts compared to water and sterile fruit were evaluated (i.e. Gasser-lure removed from analyses), there was still a significant effect of yeast type on numbers of *D. suzukii* trapped (Δ deviance = 2670, df = 6, *P* < 0.001). Post-hoc comparisons using both Dunn’s tests (with multiple testing corrections) and 95% confidence limits from the linear model revealed all traps had significantly greater numbers of *D. suzukii* than the distilled water negative control*.* In line with laboratory findings, both Dunn’s and confidence interval comparisons show *S. cerevisiae* was equally as attractive as strawberry juice. However, in contrast to laboratory assays *P. pijperi* was no more attractive than sterile juice in the field, but we note the laboratory AI corresponds with a positive capture rate in the field, and *P. pijperi* was more attractive than water in the field. *H. uvarum* (201) volatiles trapped the most *D. suzukii* in the field (mean 237), followed by *H. uvarum* (11-382) and *M. pulcherrima*, which trapped the same as each other (141 and 139 respectively; Fig. 3). However, analyses of data from the three yeasts that trapped the greatest numbers of *D. suzukii* are less clear-cut: Dunn’s adjusted comparisons indicate there is no significant difference between these three yeasts but that *H. uvarum* (201) trapped more *D. suzukii* than sterile juice (*P*_adjusted_ = 0.039) while *H. uvarum* (11-382) and *M. pulcherrima* did not (at the adjusted *P* = 0.05 level). Confidence limits derived from the linear model suggest all three yeasts trapped more *D. suzukii* than sterile juice, but that *H. uvarum* (201) trapped significantly more than *H. uvarum* (11-382) and *M. pulcherrima* (Fig. 3). Overall, the strict consensus of Dunn’s and linear model confidence limits analyses is that *H. uvarum* (201) trapped significantly greater numbers of *D. suzukii* than sterile strawberry juice in the field.

There was reasonable alignment between laboratory and field data in terms of attraction and trapping efficacy: *H. uvarum* 201, 11-382 and *M. pulcherrima* were significantly more attractive to *D. suzukii* than sterile strawberry juice in enclosed T-maze laboratory experiments and *H. uvarum* 201 trapped significantly greater numbers than sterile juice in the field, with suggestions that *H. uvarum* 11-382 and *M. pulcherrima* were also effective at trapping reasonable *D. suzukii* numbers. *S. cerevisiae* was no more attractive to *D. suzukii* than sterile fruit juice in both T-mazes and field traps but *P. pijperi* was not more attractive than sterile juice in the field. Overall, these experiments show the *D. suzukii* preference data gathered from enclosed T-mazes in the laboratory serves as a reasonable prediction for likely *D. suzukii* preferences in the field.

### Influence of yeast combinations on *D. suzukii* attraction

**Figure 4:**
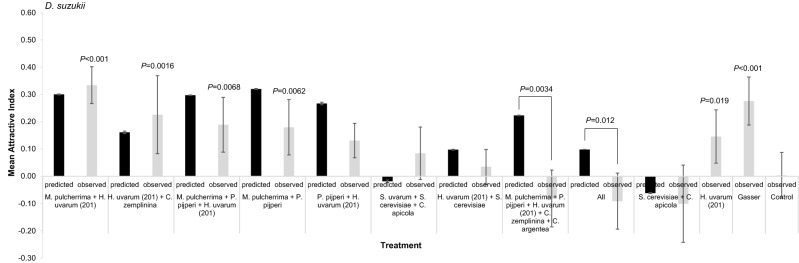
Mean (± SE) Attraction Index (AI) of *D. suzukii* to combinations of yeast metabolic volatiles (not identified) after growth in strawberry juice and then mixed in equal proportions, using laboratory T-mazes. *H. uvarum* (201), sterile fruit and commercial Gasser-lure act are controls. N = 6–7 for all treatments. Grey bars represent observed AI and black bars predicted AIs calculated from 10,000 permuted in silico mixes of corresponding individual AI values. The observed AIs of two combinations is significantly different from that predicted and are shown with solid connecting lines. P-values above bars show significance of attraction to yeast volatiles over sterile strawberry juice in the opposing T-maze arm revealed by binomial analyses.

Next, we tested how combinations of volatiles from different yeast ferments affected *D. suzukii* attraction in laboratory T-mazes. It was not possible to test all 4215 feasible combinations of 12 yeast isolates, and so we selected: (1) the three most attractive to *D. suzukii* (*M. pulcherrima* + *P. pijperi* + *H. uvarum* 201); (2) all pair-wise combinations of the three most attractive; (3) *H. uvarum* 201 + *C. zemplinina* (both attractive); (4) one attractive and one yeast *D. suzukii* was indifferent to (*H. uvarum* 201 + *S. cerevisiae*); (5) the two and three least attractive (*S. cerevisiae* + *C. apicola*; *S. cerevisiae* + *S. uvarum* + *C. apicola*); (6) all attractive yeasts with error bars that did not overlap zero (*M. pulcherrima* + *P. pijperi* + *H. uvarum* 201 + *C. zemplinina* + *C. argentea*), and finally, (7) all 12 yeasts (Fig. 4). We also included *H. uvarum* 201, sterile strawberry juice and Gasser-lure as controls. Sterile fruit juice vs sterile fruit juice controls showed no inherent bias in the T-maze apparatus (*P* = 0.43). There was significant differential attraction between the various yeast volatile combinations (Δ deviance = − 38.43, df =  − 12, *P* = 0.00013; Fig. 4). Individual binomial analyses revealed four yeast volatile combinations were significantly more attractive than sterile strawberry juice: *M. pulcherrima* + *H. uvarum* (201) (AI = 0.33, *P* = 0.000004); *H. uvarum* (201) + *C. zemplinina* (AI = 0.23, *P* = 0.0016); *M. pulcherrima* + *P. pijperi* + *H. uvarum* (201) (AI = 0.19, *P* = 0.0068); and *M. pulcherrima* + *P. pijperi* (AI = 0.18, *P* = 0.0062). *D. suzukii* was indifferent to seven yeast combinations in comparison to sterile strawberry juice. However, while a combination of volatiles from the two most attractive yeasts (*H. uvarum* 201 with *M. pulcherrima*) had the greatest AI, this combination was not significantly more attractive to *D. suzukii* than volatiles from *H. uvarum* 201 alone (equal variance *t*-test, *t* = − 1.52, *P* = 0.15).

Whether volatiles from single yeast isolates interacted in a non-additive manner for *D. suzukii* attraction when combined together was analysed with a permutation test and this revealed two combinations were significantly less attractive than predicted (Fig. 4): *M. pulcherrima* + *P. pijperi* + *H. uvarum* (201) + *C. zemplinina* + *C. argentea* (five species), and ‘all 12 yeasts’ (*P* = 0.0034 and *P* = 0.012 respectively; Fig. 4). Complementary linear model analyses also revealed that *M. pulcherrima* + *P. pijperi* + *H. uvarum* (201) + *C. zemplinina* + *C. argentea* volatile combinations differed significantly from predicted combined attractiveness (*P* = 0.0057), but the all yeast combination did not (*P* = 0.071). This indicates that some combinations of yeast volatiles may interact to result in a significant reduction in attraction, with possible repulsion, for *D. suzukii*.

### *D. suzukii* attraction to combinations of yeasts in the field

**Figure 5:**
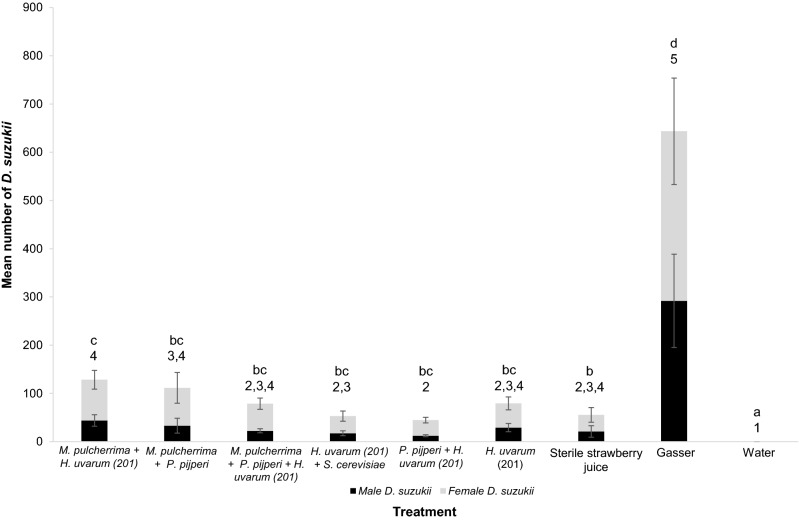
Mean numbers (± SE) of *D. suzukii* caught in field traps with various combinations of yeast metabolic volatiles (not identified) with *H. uvarum* (201), sterile strawberry juice, water and commercial Gasser-lure controls. Combinations were created by adding equal proportions after growth in strawberry juice (N = 14 except *M. pulcherrima* + *P. pijperi* N = 13). There was a significant effect of bait type on *D*. *suzukii* capture (linear model Δ deviance = 21,352, df = 8, *P* < 0.001; Kruskal–Wallis, chi-squared = 66.98, df = 8, *P* = 1.95^–11^). Two post-hoc analysis methods were employed: letters above bars connect treatments that are not significantly different using Dunn’s comparisons with Benjamini–Hochberg adjusted *P* values and numbers above bars connect treatments with overlapping 95% confidence intervals calculated from Estimated Marginal Means.

Field experiments testing combinations of ferment products that were attractive in T-maze tests show 81% of the 20,460 trapped *Drosophila* were *D*. *suzukii*. There was a significant effect of bait type on *D*. *suzukii* capture (linear model Δ deviance = 21,355, df = 8, *P* < 0.001; Kruskal–Wallis, chi-squared = 66.98, df = 8, *P* = 1.95^–11^) and Gasser-lure trapped the greatest numbers (Fig. 5). There was a significant differential effect of yeast volatile type on *D. suzukii* capture when Gasser-lure was removed from analysis (Δ deviance = 3156, df = 7, *P* < 0.001). Adjusted Dunn’s and linear model 95% confidence interval comparisons showed all baits trapped significantly greater numbers of *D. suzukii* than distilled water (Fig. 5). However, the comparison of *D*. *suzukii* trap counts between sterile fruit juice and yeast volatiles is not clear-cut. Dunn’s comparisons reveal that only the *M. pulcherrima* + *H. uvarum* 201 combination trapped significantly greater number of *D. suzukii* than the strawberry juice bait (*P*_adjusted_ = 0.023) but there were no significant differences between the remaining yeast baits, including *H. uvarum* 201 in isolation, and sterile juice or any significant differences in the numbers of *D*. *suzukii* trapped among all yeast baits (Fig. 5). Linear model 95% confidence interval comparisons indicate that no yeast bait trapped significantly more *D*. *suzukii* than sterile juice (Fig. 5). The consensus is that while the numbers of *D. suzukii* trapped by the *M. pulcherrima* + *H. uvarum* 201 combination were greater than those trapped by *H. uvarum* 201 separately, this is not statistically significant (at adjusted *P* < 0.05). Moreover, adjusted Dunn’s comparisons suggest that the *M. pulcherrima* + *H. uvarum* 201 combination trapped significantly greater numbers of *D. suzukii* than sterile strawberry juice with only a modest adjusted *P*-value (*P*_adjusted_ = 0.023).

## Discussion

We found support for the first hypothesis, that some yeast species produce specific metabolic volatiles that are more attractive to *D. suzukii* than to other *Drosophila* species, as *H. uvarum* was the only yeast species to be significantly attractive to *D. suzukii* compared to sterile fruit and was not significantly attractive to the other *Drosophila* species in laboratory assays. However, the size of the significance and effect was not large but is in line with other studies reporting *H. uvarum* to be attractive to *D. suzukii*^18,22,27,28^. *H. uvarum* was not significantly attractive to *D. melanogaster* in this study which is not in line with previous studies^15,17^. However, while the magnitudes of *P*-values vary, we note that the size and direction of attraction are consistent between the data here and that reported by Günther et al*.*^15^ for *H. uvarum* grown in strawberry juice for attraction to both *D. melanogaster* and *D. simulans* (both studies report AIs of approximately + 0.25 for *D. melanogaster* and − 0.05 for *D. simulans*). *M. pulcherrima* and *P. pijperi* were more attractive than *H. uvarum* (201) for *D. suzukii* in the laboratory, and *M. pulcherrima* and *P. pijperi* were also attractive for the *D. melanogaster* line we used. We are not aware of any previous reports showing *M. pulcherrima* and *P. pijperi* are attractive to *D. suzukii*. No one yeast isolate was significantly attractive to all three *Drosophila* species which reflects recent work suggesting there may be differences in yeast preferences between *Drosophila* species^15^. The finding that several yeast species are attractive to *D. suzukii* is in line with data from Scheidler et al*.*^18^ who found both *H. uvarum* and *C. zemphilina* to be attractive. The correspondence of *D. suzukii* attraction to volatiles from the same yeasts in laboratory and field assays suggests the two-way T-maze choice system is a reasonable approximation of *D. suzukii* responses in the field. However, it is clear this is not a perfect map as there were also differences in *D. suzukii* response. There is a range of aspects to consider when translating laboratory to field responses, including the fact that the range of other olfactory signals in the field will be greater and the effects of concentration gradients and air currents are more acute in the field. Lastly, laboratory tests were conducted with an Italian laboratory-reared strain, whereas the field tests trapped UK wild *D. suzukii*. Thus, any promising agents identified in laboratory assays should be considered putative until validated in the field.

The second hypothesis—volatiles from different genotypes of *H. uvarum* vary in their attractiveness to *D. suzukii*—must formally be rejected as there was no statistically significant difference in attraction among the eight *H. uvarum* isolates. However, there was significant attraction to volatiles from six of the eight *H. uvarum* isolates compared to sterile juice whereas the remaining two isolates were not more attractive than sterile fruit, implying there may be ecologically relevant variance between *H. uvarum* genotypes. In line with laboratory observations, *H. uvarum* (201) trapped more *D. suzukii* than *H. uvarum* (11-382) in the field and confidence interval analysis suggests this is significant (Fig. 3). Overall, the field assays are in line with laboratory data and suggest volatiles from *H. uvarum* (201 and 11-382) and *M. pulcherrima* are attractive to *D. suzukii* and provide reasonable evidence that at least these isolates are worth pursuing for *D. suzukii* control strategies.

The last hypothesis—that combinations of volatiles from different yeast species are more attractive to *D. suzukii* than volatile profiles from single yeast species—may not be accepted given these data. Four combinations of volatiles from different yeasts were more attractive than sterile juice in the laboratory, and while the *M. pulcherrima* + *H. uvarum* (201) combination reported the greatest AI (Fig. 4), this was not significantly greater than the AI from *H. uvarum* (201) separately (*P* = 0.15). We note that in the laboratory the *M. pulcherrima* + *H. uvarum* (201) combination had a greater AI than the commercial Gasser-lure bait but this did not translate to the field where the Gasser-lure bait trapped at least two times more *D. suzukii* than any yeast bait tested here. This perhaps indicates that the extent of *D. suzukii* attraction to yeasts in the field may be further optimised by altering the concentrations of volatiles released by yeast during fermentation^40,41^; here we only tested one concentration. However, the various yeast field traps were able to trap a large number of *D. suzukii* (~ 7000), and the observations that the *M. pulcherrima* + *H. uvarum* (201) bait combination trapped the greatest numbers of wild *D. suzukii* among the yeast baits and was the only yeast bait that trapped significantly greater numbers than the sterile fruit by Dunn’s comparisons indicates there may be value in exploring combined yeast baits further, particularly those involving *M. pulcherrima* and *H. uvarum* (201).

There were two yeast combinations that had significantly lower AIs than predicted based on an additive interaction of their separate volatile attraction components. The reason behind this nonlinear interaction is unknown and might be because some volatiles have a masking effect which here resulted in combinations that were less attractive, and possibly repulsive, to *D. suzukii*. This masking concept has experimental support as some volatiles act antagonistically to reduce CO_2_ repulsion^42^. The observation made here may be valuable in the development of ‘push–pull’ control strategies; for example, if these yeast combinations were not harmful to crops, then they may be deliberately added to deter *D. suzukii*. This represents a potentially significant finding in this respect, but this study focussed on attraction and not repulsion, so we only validated putatively attractive mixed yeast volatile combinations in the field.

There are clearly many other factors potentially affecting *D. suzukii* attraction that need to be considered and explored in this biologically complex tripartite yeast:fly:fruit system^15^. First, within yeast species variance for *Drosophila* attraction requires consideration: other studies that have evaluated this subject show a large range in attraction among *S. cerevisiae, S. bayanus, S. uvarum* and *S. paradoxus* isolates to *D. simulans*, *D. melanogaster* and *D. suzukii*^14,17,27^. The phenotypic range of metabolic volatile production in yeasts is a product of a genotype x environment interaction, and there is good evidence for large genetic variance within yeast species (e.g.^43–45^); such variance in attraction is likely to hold for most yeast species. The salient point is that it is probably not robust to assume the attractiveness of individual yeast genotypes are representative of attractiveness for that yeast species as a whole. We acknowledge the use of a single *D. suzukii* line in laboratory experiments here—the extent to which these observations hold in *D. suzukii* with different genetic backgrounds remains to be tested. However, the field assays involved wild populations of *D. suzukii* likely with a range of different genetic backgrounds. Another factor that may affect attraction, especially in the field, is the physiological state of the flies and the time of year. Shearer et al*.*^46^ report that by December 100% and 95% of females and males respectively are winter morphs in comparison to 50% and 30% in October in the USA. Fly fecundity also changes throughout the season, with female *D. suzukii* fecundity reduced at low temperatures^47,48^ which may affect attraction to yeast as they are important for both oviposition and egg development in *D. suzukii*^13,49^. Changes in morph and fecundity may provide part of the explanation for the change in the extent of attractiveness to the same yeasts across these field experiments spanning late October to early December (*H. uvarum* 201 baits caught half as many *D. suzukii* in the December than October traps). Another factor likely to affect attraction is fruit substrate. Strawberry was used here but Günther et al*.*^15^ show an effect of fruit type on yeast attraction to isolates of *D. simulans.* In contrast to findings in this study Scheidler et al*.*^18^ and Lasa et al*.*^27^ demonstrated that *S. cerevisiae* was attractive to *D. suzukii,* but Scheidler et al*.*^18^ used Potato Dextrose Broth and Lasa et al*.*^27^ used sucrose. Additionally, in laboratory tests certain *H. uvarum* and *S. cerevisiae* strains, derived from fruit, were significantly more attractive than a commercial *S. cerevisiae* strain when grown in corn syrup media but not when grown in sucrose-based media^27^.

These complicating factors, and many others, need exploring in efforts to use and optimise any yeast-based *D. suzukii* controls. For example, the stability of attractiveness is uncertain; 10 generations of selection on a *D. simulans* population for attraction to an unattractive yeast was sufficient to increase attraction showing *Drosophila* may be plastic over ecological time scales for attraction to yeast volatiles^15^. One last aspect to consider is that we created combinations of yeast volatiles by post-ferment blending in equal parts, and while this represents an important step in testing the attractiveness of yeast in a more ecologically realistic manner, this does not capture the true complexity of the microbial ecology on fruit as *Drosophila* attraction (at least *D. melanogaster* and *D. simulans*) is driven by volatiles in a concentration and background dependent manner^34^. Moreover, there is good evidence to show that it is the act of yeast growing together, as opposed to post-growth blending, that may produce synergistic metabolic interactions in terms of volatiles^50^, and this concept is in line with the observation that growing *S. cerevisiae* with bacteria was more attractive to *D. melanogaster* than post-growth blending^51^.

Yeast are candidates for creating attractive and selective baits for *D. suzukii* not just for monitoring and mass-trapping purposes but also in attract-and-kill strategies using phagostimulatory baits^28,52^. We have identified isolates from two species which are attractive in isolation and when combined, and this finding may contribute to developing sustainable lower insecticide input horticulture management controls for a major economically damaging pest of fruit crops.

## Materials and methods

### Drosophila cultures

*Drosophila melanogaster* were standard Oregon R wild-type (Carolina), *D. simulans* lines derived from a wild population collected from vineyards in New Zealand^14^ and *D. suzukii* cultures derived from an Italian strain, collected near Trento in 2013. All *Drosophila* species were maintained at the same temperature and light regime, 25 ± 2 °C and a 16:8 h light: dark photoperiod^53^ and reared on *Drosophila* Quick Mix Medium blue (Blades Biological Ltd.) sprinkled with dried baker’s yeast (Blades Biological Ltd.). *D. suzukii* were also cultured on media comprising 1% agar, 9% sugar, 9% pre-cooked ground maize, 2% baker's yeast, 5% malt, 1% soy flour, 0.3% propionic acid, and 0.3% methyl 4-hydroxybenzoate pre-dissolved in 70% ethanol^53^. Summer morph *D. suzukii* were housed in BugDorm cages (32.5 × 32.5 × 32.5 cm) (MegaView Science Co., Ltd.). Damp absorbent paper was placed on the base and roof of the cages to provide humidity (average 96%) inside cages. *D. suzukii* were provided with frozen raspberries weekly^53^. *D. melanogaster* and *D. simulans* were housed in standard *Drosophila* tubes (35 ml) (Gosselin FLY35-02, Fisher Scientific).

### Yeast cultures

All yeast isolates derived from the Goddard culture collection at University of Lincoln apart from *H. uvarum* 11‐382 and the origin of strains is shown in Supplementary Material, Table S1.

### Yeast volatile preparation

All yeast isolates were transferred from – 80 °C glycerol stocks to 50 ml falcon tubes containing 15 ml of YPD (1% yeast extract, 2% peptone, and 2% dextrose) media, and incubated at 30 °C for 24 h, and the optical density (600 nm) was ascertained at 24 h using a spectrophotometer (Jenway 6705). For laboratory assays, strawberry juice (var. Rociera) was sterilised by 0.2 μm filtration (Corning 1 L Filter System). For field trials, strawberry juice comprised either single or mixed varieties (Elsanta, Murano and a proprietary June bearer), and was sterilised using up to a maximum of 1 ml dimethyl dicarbonate (DMDC) dissolved in ethanol at a ratio of 1:2 DMDC to ethanol per litre of juice. This process was repeated up to three times in an attempt to sterilise the juice, after which it contained approximately less than 18 colony-forming units per ml. Strawberry juice was inoculated with 1 × 10^6^ yeast cells per ml and incubated at 30 °C for 48 h. The brix and optical density of the yeast ferments were determined at 48 h (Brix was measured using a refractometer HI 96801, Hanna Instruments) after which the juice was centrifuged at 4500 rpm for 10 min to collect cells; the supernatant containing yeast metabolites was decanted and stored frozen prior to use in both T-maze and field assays.

### Laboratory choice tests

Two-way choice test experiments were conducted to test: (1) attractiveness of yeast volatiles from 12 single yeasts to *D. suzukii*, *D. melanogaster* and *D. simulans;* (2) attraction of *D. suzukii* to volatiles from eight different strains of *H. uvarum;* and (3) attraction of *D. suzukii* to combinations of yeast volatiles. Choice tests were conducted using horizontally orientated T-maze apparatus with no forced air flow following Palanca et al*.*^17^ as shown in Fig. S1 (supplementary material). Clear Perspex vials (50 ml) were attached to the end of the arms and contained yeast volatiles or sterile strawberry juice. Damp absorbent paper was also placed in the centre of the T-maze to increase humidity for *D. suzukii*. T-maze arms contained either 10 ml of yeast ferment or 10 ml of sterile fruit juice control both at 1:1000 dilution. Where combinations of different yeasts volatiles were tested, yeasts were fermented separately, and the supernatants subsequently combined in equal proportions.

Stock fly populations were anaesthetised using CO_2_ for a maximum of six minutes to isolate females which were then starved for 24 ± 1 (*D. melanogaster* and *D. simulans*) or 17 ± 1 h (*D. suzukii*, as mortality was excessive with 24 h starvation) prior to experimentation. Flies were anaesthetised briefly using CO_2_ before being introduced into the centre of the T-maze apparatus. Between 60 and 80 mated adult females between 3 and 12 days old were added to each T-maze. Females were considered mated as they had access to males until they were sexed and then separated; mating generally occurs 1 day post-eclosion^54^. To control for any temporal effects and variability in flies’ physiological status the same cohort of flies was exposed simultaneously to all yeast treatments to constitute one replicate and this was repeated multiple times for replication. All assays were conducted with six to eight replicates in the dark to ensure that choice was driven primarily by olfactory cues. After 30 min the sliding doors of the T-maze were closed, and the T-maze placed at − 20 °C to euthanise flies prior to counting. Yeast species attraction was calculated using an Attraction Index^17^ (AI = (number of flies in yeast arm – number of flies in control arm)/total number of flies making a choice).

### Field trials

Field experiments were conducted at a commercial fruit producer’s site in Kent, UK. Drososan field traps (Koppert Biological Systems) with a 200 ml drowning solution of either single or combinations of yeast ferments after growth in strawberry juice were employed. Yeast ferments were prepared as for laboratory studies with equal volumes of separately fermented yeasts combined when required.

Three control treatments were included: strawberry juice with no yeast; distilled water (negative control); and commercially available Gasser-lure (RIGA) (positive control). Triton × 100 (0.005%, Sigma-Aldrich) was added to all drowning solutions to reduce surface tension. Traps were arranged in a randomised block design where one trap from each treatment was present in a random order per block. For the single yeast volatile trials, traps were approximately 3 m apart and 1 m from the ground in native hedgerow approximately 5 m from a raspberry crop (Fig. S2). In the combined yeast volatile trial, a second location was included along with the hedgerow set-up, and traps were placed in a deciduous bramble woodland adjacent to the hedgerow approximately 7–8 m apart and 1 m from the ground. Trials occurred across October–December 2018, and trap contents were filtered through muslin after 72 h and numbers of male and female *D. suzukii* and other *Drosophila* species were determined.

### Statistical analysis

T-mazes where no flies made a choice (i.e. no flies left the central compartment of T-maze) were omitted from analyses. Differences in attractiveness between yeast isolated were analysed separately for each *Drosophila* species using a binomial logistic regression, with treatment as a fixed factor, and significance assayed using ANOVA following model simplification as per Crawley^55^. Binomial analyses were employed to test if the sum of choices of flies from replicated tests with each yeast treatment separately were different from that expected if flies chose randomly^14,15,17,34^. We did not use the binomial tests to make comparisons between different yeast treatments and so a multiple comparison correction was not required here. Two-way ANOVA was used to determine if there was an effect of *Drosophila* and yeast species and interactions between them on AI overall the data. Permutation analysis was used to test whether volatiles from different yeast species interacted linearly or non-linearly in terms of attraction when mixed. The predicted AI of volatile combinations was based on an additive (linear) model and was compared to the observed AI from volatile combinations. The null/additive distribution of combined AI values for each of the volatile combinations were created by randomly selecting AI values from single yeasts among respective replicates for each of the constituent yeasts and calculating the mean and repeating 10,000 times for each. The experimentally observed AI values from combinations were compared to this null distribution. A linear model was also used to analyse attraction to combinations of yeast species volatiles to test whether yeasts interacted linearly or non-linearly (Fig. S3), and whether flies’ preference differed between the combination and their constituent yeasts separately was captured in the coefficients (y = Xβ + ε). Student’s *t*-tests were used to determine if the most significantly attractive combined yeast volatiles were more attractive than the volatiles from *H. uvarum* 201 alone. Field trap capture data was analysed using non-parametric rank based Kruskal–Wallis tests with Dunn’s post-hoc comparisons with *P*-values adjusted with the Benjamini–Hochberg method, and generalised linear models with a Poisson error structure where treatment and sex were treated as fixed factors and block as a random effect. All statistical analyses were carried out in R version 3.6.1^56^ and the lme4^57^ package was used for the binomial logistic regression and linear regression and emmeans^58^ and Hmisc^59^ packages were used to generate confidence intervals.

### Ethics approval

This project was approved by the University of Lincoln ethics board (CoSREC388).

## Supplementary Information

Supplementary Information 1.

Supplementary Information 2.

Supplementary Information 3.

## Data Availability

All raw data and code are available in the supplementary material.
