# Supplementary material for: Separate and combined Hanseniaspora uvarum and Metschnikowia pulcherrima metabolic volatiles are attractive to Drosophila suzukii in the laboratory and field
Source: Sci Rep. 2021 Jan 13;11:1201. doi: 10.1038/s41598-020-79691-3 (PMC7806593; doi:10.1038/s41598-020-79691-3)
Supplement: Supplementary file 1 — Supplementary Information 1. [file 41598_2020_79691_MOESM1_ESM.pdf]

Supplementary material: Separate and combined *Hanseniaspora uvarum* and *Metschnikowia pulcherrima* metabolic volatiles are attractive to *Drosophila suzukii* in the laboratory and field

Jones R.[1,2]*, Fountain M.T.[2], Günther C.S.[1,3], Eady P.E.[1] and Goddard M.R.[1,4]

[1]School of Life Sciences, University of Lincoln, Lincoln, LN6 7DL, UK.

[2]NIAB EMR, New Road, East Malling, Kent ME19 6BJ, UK.

[3]Current address: The New Zealand Institute of Plant and Food Research Ltd, Albert Road, Auckland 1025, Auckland, New Zealand.

[4]The School of Biological Science, University of Auckland, New Zealand

*Corresponding author: roryjones@lincoln.ac.uk

Table S1: Origin, source and strain of yeast isolates used in this study.

Species	Strain	Origin	Source	Reference
Saccharomyces cerevisiae	EC-1118	France	Commercial wine yeast	Lallemand Inc.
Pichia kluyveri	162	New Zealand	Chardonnay juice	Anfang et al. 2009
Pichia pijperi	218	New Zealand	Pinot noir ferment	Goddard culture collection
Candida apicola	150	New Zealand	Beehive	Anfang et al. 2009
Candida zemplininia	164	New Zealand	Chardonnay ferment	Anfang et al. 2009
Hanseniaspora occidentalis	212	New Zealand	Syrah fruit	Gayevskiy et al. 2012
Saccharomyces uvarum	198	New Zealand	Sauvignon Blanc ferment	Goddard culture collection
Metschnikowia pulcherrima	190	New Zealand	Sauvignon Blanc ferment	Goddard culture collection
Torulaspora delbrueckii	166	New Zealand	Sauvignon Blanc ferment	Goddard culture collection
Candida argentea	98-3	United Kingdom	*D. subobscura*	Goddard culture collection
Pichia nakasei	44-1	United Kingdom	*D. subobscura*	Goddard culture collection
Hanseniaspora uvarum	201	New Zealand	Chardonnay fruit	Gayevskiy et al. 2012
Hanseniaspora uvarum	206	New Zealand	Chardonnay fruit	Goddard culture collection
Hanseniaspora uvarum	209	New Zealand	Chardonnay fruit	Goddard culture collection
Hanseniaspora uvarum	11-382	United States	*D. suzukii*	Phaff Yeast culture collection, UC-Davis
Hanseniaspora uvarum	44-9	United Kingdom	*D. subobscura*	Goddard culture collection
Hanseniaspora uvarum	28-1	United Kingdom	*Drosophila* sp.	Goddard culture collection
Hanseniaspora uvarum	28-5	United Kingdom	*Drosophila* sp.	Goddard culture collection
Hanseniaspora uvarum	28-9	United Kingdom	*Drosophila* sp.	Goddard culture collection

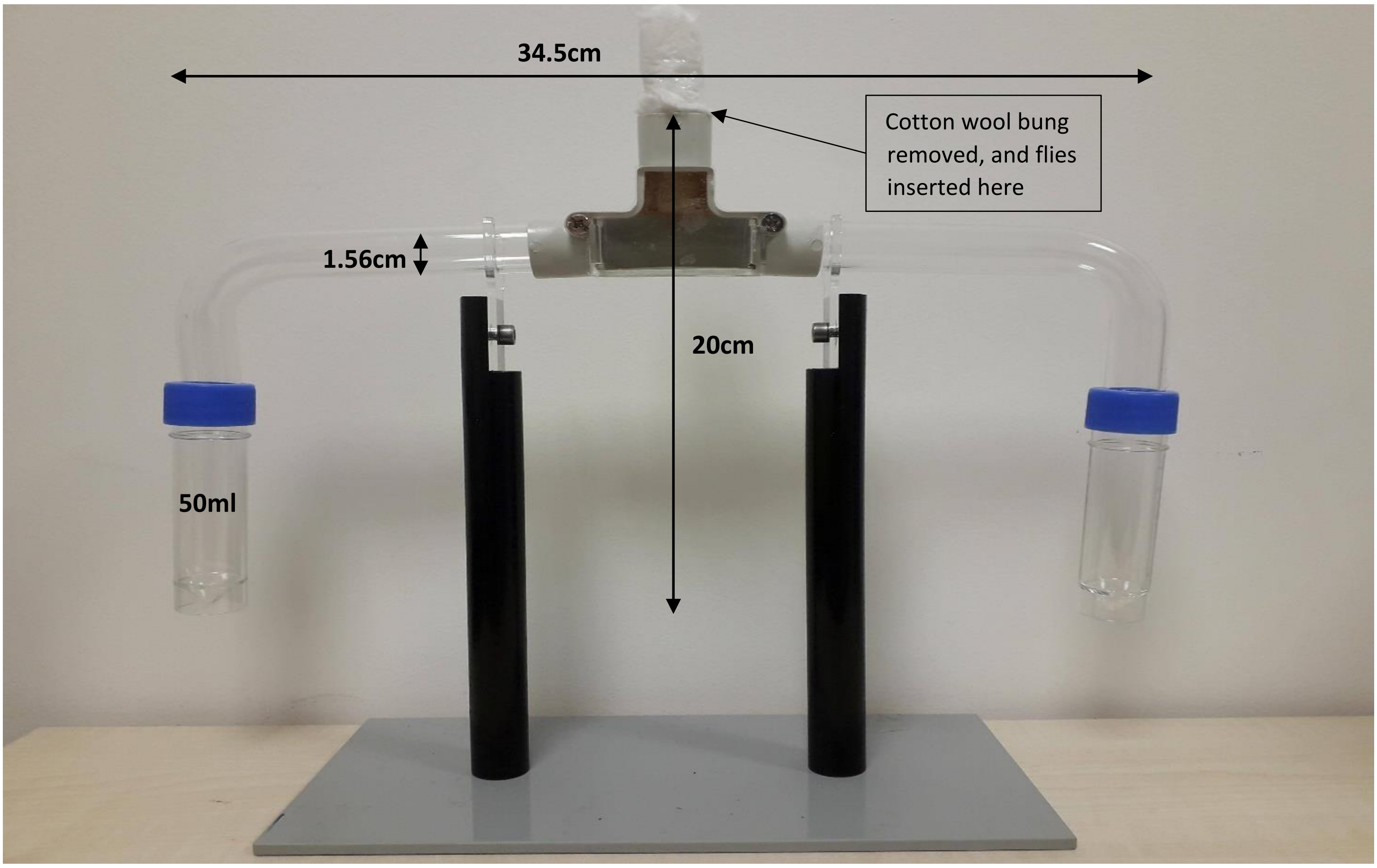

Fig. S1 Set up of T-maze apparatus with dimensions. 10 ml of 1:1000 dilution of yeast ferment or sterile fruit juice for a control was placed in the vials attached to the T-maze arms, a piece of circular mesh was placed in between the top of the vial and the end of the T-maze arms to prevent *Drosophila* interacting with the ferments/fruit juice whilst simultaneously allowing diffusion of odours throughout the T-maze. For all experiments using *D. suzukii* a damp blue absorbent paper was included in the centre of the T-maze to increase humidity. *Drosophila* were placed in the T-maze after being anaesthetised. All assays were conducted in the dark to prevent remove the influence of visual cues, and 60-80 starved mated adult females between 3-12 days old were added to each T-maze for 30 mins before recording choice.

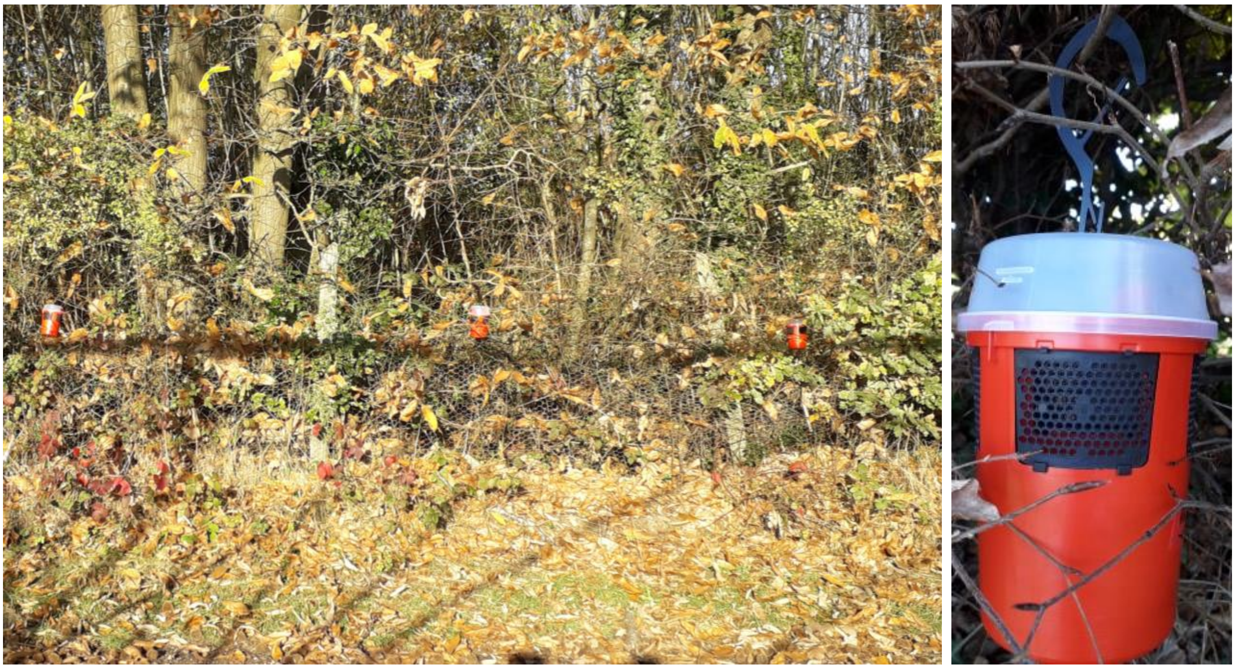

Fig S2. Set up of Drososan traps (Koppert Biological Systems) in a native hedgerow approximately 5m from a raspberry crop. Traps contained 200 ml drowning solution of either separate or combinations of yeast ferments after growth in strawberry juice, as well as three control treatments: strawberry juice with no yeast; distilled water (negative control); and commercially available Gasser-lure (RIGA) (positive control). Traps placed approximately 3 m apart and 1 m from the ground, and arranged in a randomised block design where one trap from each treatment was present in a random order per block. In the combined yeast volatile trial a second location was also included along with the hedgerow set-up, and traps were placed in a deciduous bramble woodland adjacent to the hedgerow approximately 7-8 m apart and 1 m from the ground (not shown).

To answer this question, we fitted a standard linear model,

$$\mathbf{y} = \mathbf{X}\beta + \epsilon \tag{1}$$

where the parameter vector β is composed of two vectors, $\beta = (\beta_s, \beta_c)^\top$, and where β_s represents the true population AI for each single yeast, and β_c represents an offset AI for each combination, compared with that predicted by the individual yeasts' AIs. Specifically, the AI for a given combination i is defined to be

$$\mathrm{AI}_i = \mathbf{c}_i^\top \beta_s + \beta_{ci}, \tag{2}$$

where the elements of $\mathbf{c}_i$ represent the proportions of the respective single yeasts in the combination.

These parameters were estimated using a linear model, with a design matrix again composed of two parts,

$$\mathbf{X} = \begin{bmatrix}\mathbf{S} & \mathbf{C}\end{bmatrix} \tag{3}$$

Each row in $\mathbf{X}$ represents a given replicate. The elements on a given row of $\mathbf{S}$ represents the composition of the yeast for that replicate as proportions of the single yeasts:

$$\mathbf{S} = \begin{bmatrix} 1 & 0 & 0 & 0 & \dots & 0 \\ 1 & 0 & 0 & 0 & \dots & 0 \\ \vdots & \vdots & \vdots & \vdots & & \vdots \\ 0 & 1 & 0 & 0 & \dots & 0 \\ 0 & 1 & 0 & 0 & \dots & 0 \\ \vdots & \vdots & \vdots & \vdots & & \vdots \\ 0 & 0 & 0 & 0 & \dots & 1 \\ \vdots & \vdots & \vdots & \vdots & & \vdots \\ \frac{1}{2} & \frac{1}{2} & 0 & 0 & \dots & 0 \\ \frac{1}{2} & \frac{1}{2} & 0 & 0 & \dots & 0 \\ \vdots & \vdots & \vdots & \vdots & & \vdots \\ \frac{1}{12} & \frac{1}{12} & \frac{1}{12} & \frac{1}{12} & \dots & \frac{1}{12} \end{bmatrix} \tag{4}$$

(Here, the first two rows represent replicates with pure samples of the first yeast, and the last row represents a replicate with the equal combination of all twelve yeasts).

This then allows the AIs for the combination yeasts to be modelled as linear mixtures of the AIs of the pure yeasts (mixed in the same proportions as the yeasts themselves). The parameters β_c then allow the true AIs of the combination yeasts to deviate from these mixed values. To capture this, the other part of the design matrix $\mathbf{C}$ contains flags that are 0 or 1, being 1 only in column i if the yeast is an example of mixture i. Note that for rows corresponding to replicates with the pure yeasts, all elements are zero.

$$\mathbf{C} = \begin{bmatrix} 0 & 0 & 0 & 0 & \dots & 0 \\ 0 & 0 & 0 & 0 & \dots & 0 \\ \vdots & \vdots & \vdots & \vdots & & \vdots \\ 1 & 0 & 0 & 0 & \dots & 0 \\ 1 & 0 & 0 & 0 & \dots & 0 \\ \vdots & \vdots & \vdots & \vdots & & \vdots \\ 0 & 1 & 0 & 0 & \dots & 0 \\ 0 & 1 & 0 & 0 & \dots & 0 \\ \vdots & \vdots & \vdots & \vdots & & \vdots \\ 0 & 0 & 0 & 0 & \dots & 1 \end{bmatrix} \tag{5}$$

Fig. S3 Linear model used to test whether the preferences for the combinations of yeast differed from that predicted based on a linear mixture of the preferences of individual yeasts.

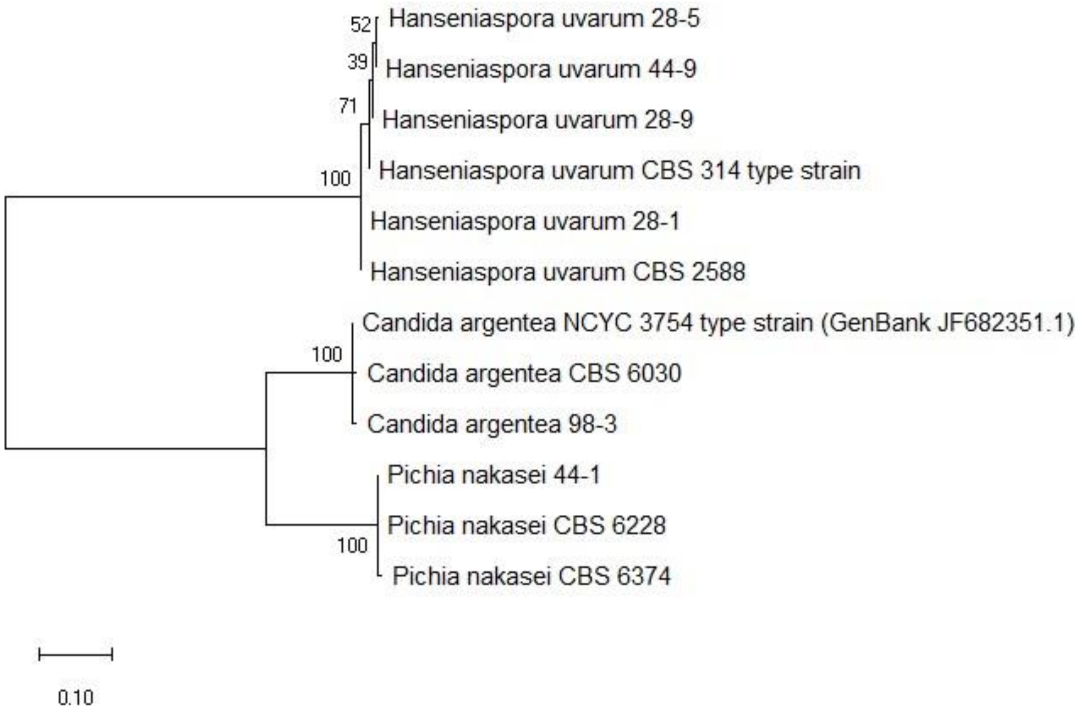

Fig S3. Maximum likelihood tree of the sanger sequences isolates and reference species. For the reference species, were possible ITS sequences of type strains taken from the CBS culture collection website were used. If none were available other strains with ITS sequences available were included. Additionally, sequences taken from Genbank were also used in some cases (accessed using blast search). Sequences from the reference species were then aligned one by one using cluster omega, with the corresponding sanger sequences trimmed to the same length. All the sequences were then aligned in Mega x and a Maximum likelihood tree with bootstrapping (500) was generated in Mega x.

Output of post-hoc field trap *D. suzukii* number comparison analyses

1. Comparisons of separate yeast

Treatments:
1= Distilled water
2= Sterile strawberry juice
3= *H. uvarum* 201
4= *H. uvarum* (11-382)
5= *M. pulcherrima*
6= *P. pijperi*
7= *S. cerevisiae*
8= Gasser-lure

```
Kruskal-Wallis rank sum test

data:  Number of D. suzukii by Treatment
Kruskal-Wallis chi-squared = 41.593, df = 7, p-value = 6.229e-07


Dunn (1964) Kruskal-Wallis multiple comparison   p-
values adjusted with the Benjamini-Hochberg method.

   Comparison           Z      P.unadj        P.adj
1       1 - 2 -1.70227665 8.870352e-02 1.460999e-01
2       1 - 3 -4.19894908 2.681566e-05 3.754193e-04
3       2 - 3 -2.49667242 1.253647e-02 3.900235e-02
4       1 - 4 -3.48708793 4.883108e-04 3.418175e-03
5       2 - 4 -1.78481128 7.429193e-02 1.485839e-01
6       3 - 4  0.71186115 4.765508e-01 5.559759e-01
7       1 - 5 -3.30138502 9.620876e-04 3.848350e-03
8       2 - 5 -1.59910837 1.097965e-01 1.618054e-01
9       3 - 5  0.89756405 3.694180e-01 4.497263e-01
10      4 - 5  0.18570291 8.526777e-01 8.842584e-01
11      1 - 6 -1.75386079 7.945436e-02 1.483148e-01
12      2 - 6 -0.05158414 9.588601e-01 9.588601e-01
13      3 - 6  2.44508828 1.448167e-02 4.054867e-02
14      4 - 6  1.73322714 8.305531e-02 1.453468e-01
15      5 - 6  1.54752423 1.217369e-01 1.704316e-01
16      1 - 7 -1.17611841 2.395476e-01 3.193967e-01
17      2 - 7  0.52615824 5.987783e-01 6.448381e-01
18      3 - 7  3.02283066 2.504223e-03 8.764782e-03
19      4 - 7  2.31096952 2.083454e-02 5.303337e-02
20      5 - 7  2.12526661 3.356437e-02 7.831687e-02
21      6 - 7  0.57774238 5.634381e-01 6.310506e-01
22      1 - 8 -5.17904775 2.230214e-07 6.244599e-06
23      2 - 8 -3.47677110 5.074909e-04 2.841949e-03
24      3 - 8 -0.98009868 3.270374e-01 4.162294e-01
25      4 - 8 -1.69195982 9.065363e-02 1.410168e-01
26      5 - 8 -1.87766273 6.042732e-02 1.301511e-01
27      6 - 8 -3.42518696 6.143765e-04 2.867090e-03
28      7 - 8 -4.00292934 6.256299e-05 5.839213e-04
```

Confidence limits numbers from analyses:

```
TreatmentWith2AsRef emmean     SE  df asymp.LCL asymp.UCL
 2                   53.30 1.10 Inf      44.26    64.39
 1                    0.65 1.66 Inf      0.24     1.76
3                   232.06 1.09 Inf      196.37   273.14
4                   138.80 1.09 Inf      116.75   164.19
5                   136.18 1.09 Inf      115.58   161.26
6                   56.26 1.10 Inf      46.53    67.83
7                   40.21 1.11 Inf      33.12    49.06
8                   476.75 1.08 Inf      407.48   558.92

Confidence level used: 0.95
```

2. Comparisons of combination of yeasts

1= Distilled water

2= Sterile strawberry juice

3= *H. uvarum* 201

4= *M. pulcherrima* + *P. pijperi* + *H. uvarum* 201

5= *M. pulcherrima* + *P. pijperi*

6= *M. pulcherrima* + *H. uvarum* (201)

7= *P. pijperi* + *H. uvarum* 201

8= *H. uvarum* 201 + *S. cerevisiae*

9= Gasser-lure

```
Kruskal-Wallis rank sum test

data:  Number of D. suzukii by Treatment
Kruskal-Wallis chi-squared = 66.981, df = 8, p-value = 1.956e-11


Dunn (1964) Kruskal-Wallis multiple comparison   p-
values adjusted with the Benjamini-Hochberg method.

   Comparison           Z      P.unadj        P.adj
1       1 - 2 -2.64383993 8.197141e-03 2.107836e-02
2       1 - 3 -4.35072177 1.356901e-05 8.141408e-05
3       2 - 3 -1.70688185 8.784402e-02 1.664413e-01
4       1 - 4 -4.60388315 4.146852e-06 3.732167e-05
5       2 - 4 -1.96004322 4.999074e-02 1.058627e-01
6       3 - 4 -0.25316137 8.001435e-01 8.728838e-01
7       1 - 5 -4.39173559 1.124494e-05 8.096357e-05
8       2 - 5 -1.79731760 7.228521e-02 1.445704e-01
9       3 - 5 -0.12234289 9.026275e-01 9.284168e-01
10      4 - 5  0.12608607 8.996638e-01 9.525852e-01
11      1 - 6 -5.23809154 1.622456e-07 2.920421e-06
12      2 - 6 -2.59425162 9.479708e-03 2.275130e-02
13      3 - 6 -0.88736977 3.748799e-01 5.398270e-01
14      4 - 6 -0.63420839 5.259448e-01 6.107746e-01
15      5 - 6 -0.74843906 4.541954e-01 5.839655e-01
16      1 - 7 -3.70085391 2.148752e-04 7.735506e-04
17      2 - 7 -1.05701399 2.905052e-01 4.547038e-01
18      3 - 7  0.64986786 5.157776e-01 6.189331e-01
19      4 - 7  0.90302923 3.665104e-01 5.497656e-01
```

```
20      5 - 7  0.76006262 4.472172e-01 5.962895e-01
21      6 - 7  1.53723763 1.242351e-01 2.236232e-01
22      1 - 8 -3.77915125 1.573638e-04 6.294553e-04
23      2 - 8 -1.13531132 2.562449e-01 4.193098e-01
24      3 - 8  0.57157053 5.676130e-01 6.385646e-01
25      4 - 8  0.82473190 4.095238e-01 5.670330e-01
26      5 - 8  0.68322892 4.944622e-01 6.138151e-01
27      6 - 8  1.45894029 1.445815e-01 2.478541e-01
28      7 - 8 -0.07829733 9.375915e-01 9.375915e-01
29      1 - 9 -7.64703947 2.056592e-14 7.403732e-13
30      2 - 9 -5.00319954 5.638652e-07 6.766382e-06
31      3 - 9 -3.29631770 9.796117e-04 3.206002e-03
32      4 - 9 -3.04315632 2.341107e-03 6.483065e-03
33      5 - 9 -3.11235595 1.856006e-03 5.568017e-03
34      6 - 9 -2.40894793 1.599858e-02 3.599681e-02
35      7 - 9 -3.94618556 7.940609e-05 4.083742e-04
36      8 - 9 -3.86788822 1.097820e-04 4.940188e-04
```

Confidence limits numbers from analyses:

```
TreatmentWith2AsRef emmean    SE  df asymp.LCL asymp.UCL
 2                   39.65 1.25 Inf     25.53     61.56
 1                    0.10 2.10 Inf      0.02      0.44
3                   56.83 1.25 Inf     36.60     88.23
4                   56.26 1.25 Inf     36.23     87.36
5                   83.10 1.25 Inf     54.05     129.02
6                   91.34 1.25 Inf     59.15     142.59
7                   31.82 1.25 Inf     20.49     49.40
8                   37.71 1.25 Inf     24.29     58.56
9                   459.44 1.25 Inf     295.89     713.37

Confidence level used: 0.95
```
